# The durable effect of acupuncture for episodic migraine: a systematic review and meta-analysis

**DOI:** 10.3389/fnins.2023.1211438

**Published:** 2023-09-08

**Authors:** Hangyu Shi, Runyu Miao, Shuai Gao, Lili Zhu, Jiufei Fang, Zhishun Liu

**Affiliations:** ^1^Department of Acupuncture, Guang'anmen Hospital, China Academy of Chinese Medical Sciences, Beijing, China; ^2^Graduate College, Beijing University of Chinese Medicine, Beijing, China; ^3^Institute of Metabolic Diseases, Guang'anmen Hospital, China Academy of Chinese Medical Sciences, Beijing, China

**Keywords:** acupuncture, migraine, durable effect, systematic review, RCTs

## Abstract

**Background:**

Migraine is a common and recurrent type of headache. Avoiding trigger factors is not often successful in reducing headache frequency, duration, and severity. Prophylactic medications may be effective but are limited by strict indications and daily medication intake. This review aimed to investigate the durable effect of acupuncture on episodic migraine.

**Methods:**

Seven databases including Medline, Embase, PubMed, etc., were searched for English and Chinese literature from their inception to 23 November 2022. Two independent reviewers screened the retrieved studies and extracted the data. Primary outcomes were monthly migraine days, monthly migraine attacks, and VAS score at 3 months post-treatment. The risk of bias in included studies was assessed using the Cochrane Risk of Bias 2.0 tool. Meta-analysis was conducted where applicable.

**Results:**

Fifteen studies were included in this review. Acupuncture reduced the number of migraine attacks (MD -0.68; 95% CI –0.93, −0.43; *p* < 0.001), the number of days with migraine (MD –0.86; 95% CI –1.18, −0.55; *p* < 0.001), and VAS score (MD –1.01; 95% CI –1.30, −0.72; *p* < 0.001) to a greater degree than sham acupuncture at 3 months after treatment. Significant differences in reducing pain intensity of migraine in favor of acupuncture compared with waitlist (MD –1.84; 95% CI –2.31, −1.37; *p* < 0.001) or flunarizine (MD –2.00; 95% CI –2.35, −1.65; *p* < 0.001) at 3 months after treatment were found, and the differences reached the minimal clinically important difference (MCID).

**Conclusion:**

This review found that the durable effect of acupuncture for episodic migraine lasted at least 3 months after treatment. More high-quality studies with longer follow-up periods in the future are needed to confirm the findings.

## 1. Introduction

Migraine is a disabling disorder that is typically characterized by recurrent moderate to severe attacks of headache, often lasting hours to days (Steiner et al., 2019), which is very common among all age groups and more prevalent in women than men (Stovner et al., 2022). It was ranked as the second most disabling disease worldwide with a global prevalence of 15%, associated with an annual financial burden estimated at $23 billion in the United States (Steiner et al., 2020; Ashina et al., 2021). Among migraine patients, young people reported the highest incidence rate and older adults reported the highest 1-year prevalence which increased with age. According to the International Headache Society (IHS) classification, the most frequent type of migraine is episodic migraine, with attacks occurring randomly with or without aura (Headache Classification Committee of the International Headache Society (IHS), 2018). Although there are verified migraine triggers, including stress, premenstrual periods, alcohol, and bad weather, most patients experience unpredictable attacks from month to month and fail to prevent migraine attacks by simply avoiding triggers (Marmura, 2018). The uncertainty of episodic migraine and the accompanying symptoms impair the quality of daily life and possibly lead to anxiety and depression (May and Schulte, 2016). With unsatisfied management and other risk factors, 2.5% of the patients with episodic migraine ultimately turn to chronic migraine (Andreou and Edvinsson, 2019).

The clinical recommendations for migraine consist of two situations: analgesics for acute attacks including nonsteroidal anti-inflammatory drugs (NSAIDs), aspirin, triptan, and paracetamol; and prophylactic medication including metoprolol, propranolol, flunarizine, valproic acid, and topiramate (Evers et al., 2009; Kennis et al., 2013; Tfelt-Hansen, 2013). Using analgesics does not prevent future attacks but increases the risk of chronic migraine (Su and Yu, 2018). Prophylactic drugs are carefully prescribed only for patients with frequent attacks or severe auras that significantly impair quality of life and with no contraindication. Daily intake of preventive drugs usually lasts 3–6 months protecting patients from frequent attacks (Evers et al., 2009). However, the long-term effect after drug withdrawal has rarely been studied. Both flunarizine and beta-blockers failed to maintain the success of prophylaxis with a marked decrease after treatment discontinuation (Wöber et al., 1991). Considering the huge burden of migraine, the Global Campaign Against Headache has recently emphasized that more effort is needed to reduce migraine attack frequency and duration, reduce disability, and reduce health-related distress (The American Headache Society Position Statement On Integrating New Migraine Treatments Into Clinical Practice, 2019; Olesen and Jensen, 2023).

**Figure 1:**
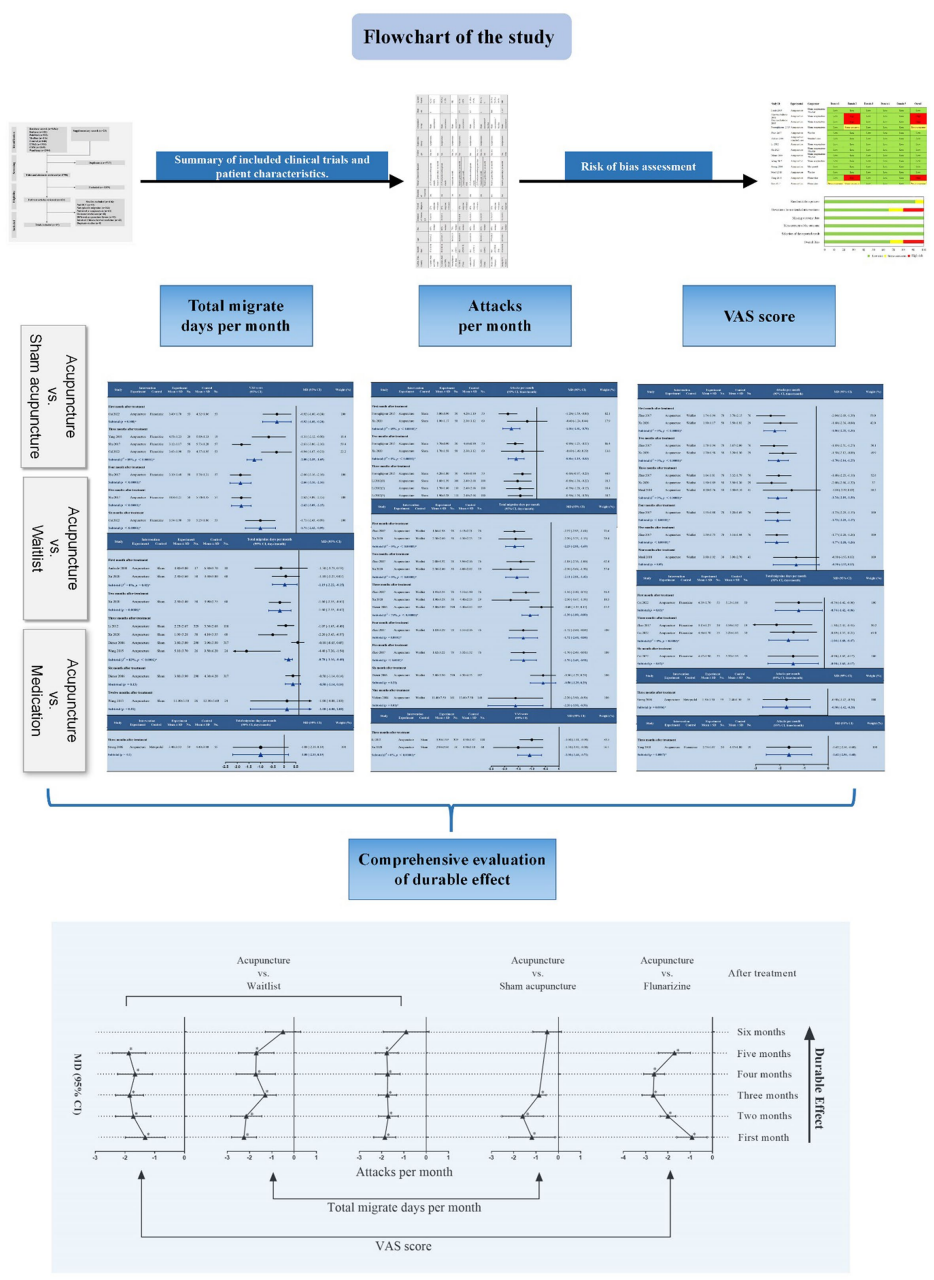
Flow chart of the study. RCT, randomized controlled trial.

Acupuncture has been recommended as an optional treatment for episodic migraine by the National Institute for Health and Care Excellence (NICE) (Kennis et al., 2013). Besides, recent reviews consistently suggested that acupuncture’s benefit for migraine is similar to preventive drugs and superior to sham acupuncture (Linde et al., 2016; Wells et al., 2019; Zhang et al., 2020). Another network meta-analysis reported that acupuncture showed a better effect than propranolol in reducing the number of migrainous attacks (Chen et al., 2020). Another notable feature of acupuncture is the durable effect after treatment, which can achieve longer-term therapeutic effects with fewer sessions, greatly reducing the burden of patients with episodic migraine. Latest clinical trials have revealed the long-lasting effect of acupuncture for episodic migraine in the post-treatment periods (Zhao et al., 2017; Xu et al., 2020) although the previous study did not find any significant difference between acupuncture and sham acupuncture in reducing migraine attacks at post-treatment follow-ups (Linde et al., 2005). However, the durable effect of acupuncture for episodic migraine has not been systematically reviewed to date. Therefore, we conducted this focused, systematic review of high-quality, randomized controlled trials (RCTs) to investigate the durable effect of acupuncture for episodic migraine after discontinuation of treatment. Figure 1 shows the flow chart of our research.

## 2. Materials and methods

We performed this systematic review in accordance with the Preferred Reporting Items for Systematic Review and Meta-Analysis (PRISMA) statement (Page et al., 2021). The protocol has been previously registered on PROSEPERO (ID: CRD42023394096).

### 2.1. Inclusion criteria

We identified relevant original studies following the PICOS principle (Table 1): Steiner et al. (2019) study type: RCTs; (Stovner et al., 2022) object of study: adult (age ≥ 18) patients with episodic migraine (with or without aura); (Ashina et al., 2021) intervention: acupuncture (including manual acupuncture, electroacupuncture, fire needling acupuncture, auricular acupuncture, scalp acupuncture, and warm needle moxibustion) compared with sham acupuncture, waitlist, or any pharmacological therapy (acute or prophylactic treatment); (Steiner et al., 2020) primary outcomes: the article must report at least one of the following primary outcomes and follow-up the patients for at least 3 months after treatment: ① total migraine days per month, ② attacks per month, ③ visual analog scale (VAS) score or numerical rating scale (NRS); [Headache Classification Committee of the International Headache Society (IHS), 2018] peer-reviewed articles that have been published in a journal; (Marmura, 2018) published in English or Chinese.

**Table 1:** Population, intervention, comparison, outcomes, and study design (PICOS) criteria for study selection.

**Parameters**	**Descriptions**
Object of study	Adult with episodic migraine.
Intervention	Acupuncture, electroacupuncture, fire needling acupuncture, auricular acupuncture, scalp acupuncture, and warm needle moxibustion.
Comparison	Sham acupuncture, waitlist, or any pharmacological therapy.
Outcome	(1) Total migraine days per month, (2) attacks per month, and (3) VAS score.
Setting	Peer-reviewed articles (RCTs) in English or Chinese.

### 2.2. Exclusion criteria

Literature with the following characteristics was excluded: Steiner et al. (2019) studies investigating the effect of acupressure, moxibustion, laser acupuncture, or acupoint injection; Stovner et al. (2022) studies including patients with chronic migraine, cluster headache, tension-type headache, or menstrual migraine; Ashina et al. (2021) studies involving Chinese herbal medicine in any group, or comparing different needle insertion sites (different acupoints) or different forms of acupuncture; Steiner et al. (2020) studies with invalid sham control (inserting needles as deeply as verum acupuncture at acupoints or non-acupoints); Headache Classification Committee of the International Headache Society (IHS) (2018) quasi-RCTs; Marmura (2018) duplicate publications; May and Schulte (2016) articles without available data or research without full text; Andreou and Edvinsson (2019) articles with unclear follow-up timepoints.

### 2.3. Literature retrieval, screening, and data extraction

The RCT search strategy published by the Cochrane Collaboration was used to perform the literature search. We searched Seven databases including Medline, Embase, PubMed, the Cochrane Central Register of Controlled Trials (CENTRAL), China National Knowledge Infrastructure (CNKI), WanFang Database, and Chinese Biomedical Literature Database (CBM) for English and Chinese literature from their inception to 23 November 2022. Please refer to Supplementary Data Sheet S1 for more information about the search strategy. We also searched references of current reviews, ClinicalTrials.gov, the WHO International Clinical Trials Registry Platform (ICTRP), and the Chinese Clinical Trial Registry (ChiCTR) for potentially eligible studies.

The retrieved literature was included in the Endnote X9 software to remove duplicates. Two researchers were assigned to double-check the retrieved literature, read the title and abstract for preliminary screening, and read the full text for further screening. Data extraction also adopted double entry and cross-checking. In case of disagreement, a third senior researcher was consulted to reach a consensus. We contacted the authors to obtain complete data for literature with incomplete data. Data extraction included title, author, number of patients, average age, gender, study type, intervention and comparison, duration of treatment, outcome measures, follow-ups, and adverse events. Both primary outcomes and secondary outcomes were extracted. Secondary outcomes included response rate, safety, disability, quality of life, and anxiety and depression.

### 2.4. Risk of bias assessment

Based on the Cochrane Risk of Bias 2.0 tool for RCTs (Sterne et al., 2019), a revised domain-based evaluation introduced by Cochrane Collaboration, two researchers independently assessed the risk of bias of included studies. This tool considered five domains of bias: randomization process, deviations from intended interventions, missing outcome data, measurement of the outcome, and selection of the reported results. The overall assessment was classified into three categories “low,” “high,” and “some concerns,” corresponding to the worst risk of bias identified across all domains.

### 2.5. Statistical analyses

RevMan (version 5.3) and Prism (version 7.0) were used to analyze the data. The effect value of categorical variables was expressed by risk ratio (RR), the effect value of continuous variables was expressed by mean difference (MD), and the 95% confidence interval (CI) was used to express the statistical analysis results. *I^2^* statistics were used to measure heterogeneity. A fixed-effect model was used if *I^2^* < 50%; otherwise, the random-effect model was used. Publication bias was explored through a funnel-plot analysis.

## 3. Results

### 3.1. Study characteristics

**Figure 2:**
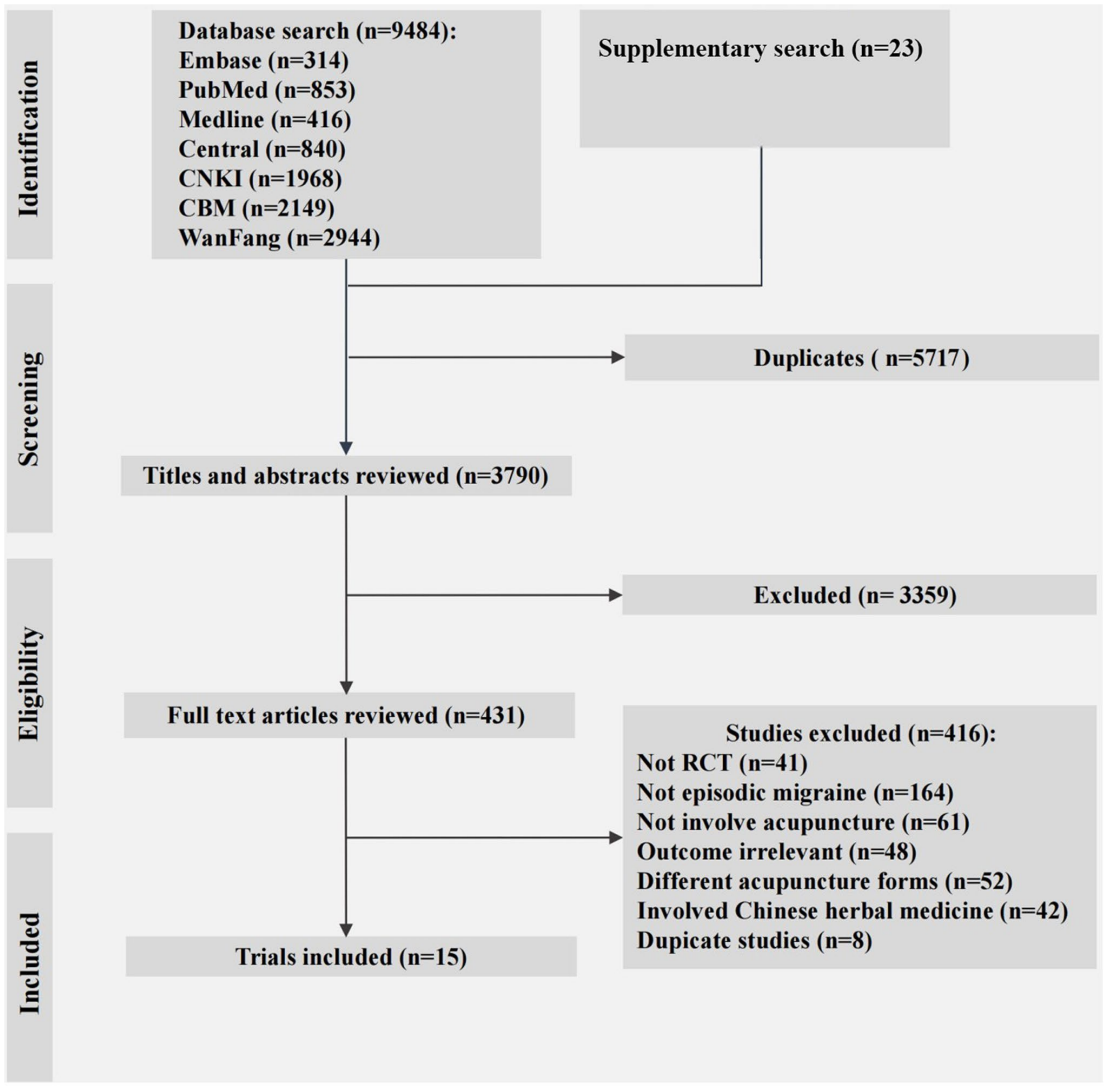
PRISMA flow diagram. RCT, randomized controlled trial.

We found 853 articles from PubMed, 314 from Embase, 416 from Medline, 840 from Central, 1968 from CNKI, 2149 from CBM, and 2,944 from WanFang. After results from these searches were combined and duplicates removed, the total number of articles was 3,790. Of these, 3,359 were excluded based on their titles and abstracts. Of the 416 that underwent full-text evaluation, 15 met the inclusion criteria and were included for analysis (Figure 2).

A total number of 3,035 patients with episodic migraine were enrolled in the 15 included studies. There was no statistical difference between the baselines of all study descriptions. Eight studies compared acupuncture with sham acupuncture (Linde et al., 2005; Alecrim-Andrade et al., 2006; Diener et al., 2006; Alecrim-Andrade et al., 2008; Li et al., 2012; Foroughipour et al., 2014; Wang et al., 2015; Xu et al., 2020), five studies compared acupuncture with waitlist (Vickers et al., 2004; Diener et al., 2006; Zhao et al., 2017; Musil et al., 2018; Xu et al., 2020), and four studies compared acupuncture with prophylactic drugs (Streng et al., 2006; Shu et al., 2017; Yang et al., 2018; Cai et al., 2022). Patients received 8–16 treatments over 4–12 weeks in most studies; the details of interventions are shown in Supplementary Data Sheet S2. Only Wang’s study (Wang et al., 2015) had a longer treatment duration, 20 weeks. We also summarized each trial’s clinical and methodological characteristics and primary outcomes (Table 2).

**Table 2:** Characteristics of included studies.

Author, Year, Country	Sample size	Age	Sex	Duration of disease (y)	Type of migraine	Duration of treatment	Primary outcome measures	Follow-ups	Experiment group	Comparison group	Drop out	Adverse events
Alecrim-Andrade 2008, Brazil	37 (19/18)	35.0 ± 9.2	89% women	17.6 ± 9.8	27% migraine with aura	3 m	≥50% reduction of migraine attacks	1 m, 6 m	Acupuncture	Sham acupuncture	1	8.2%/8.8%
							Total migraine days per month	1 m				
Alecrim-Andrade 2006, Brazil	28 (14/14)	24.7 ± 11.7	89% women	18.5 ± 8.3	21% migraine with aura	3 m	≥50% reduction of migraine attacks	6 m	Acupuncture	Sham acupuncture	0	19.6%/11.2%
Foroughipour 2014, Iran	100 (50/50)	36.5 ± 11.0	79% women	–	–	1 m	Attacks per month	1 m, 2 m, 3 m	Acupuncture	Sham acupuncture	0	NR
Li 2012, China	476 (358/118)	36.8 ± 12.2	59% women	8.2 ± 0.4	12% migraine with aura	1 m	Total migraine days per month	3 m	Acupuncture	Sham acupuncture	37	8.1%/6.8%
							Attacks per month					
							VAS					
Linde 2005, Germany	226 (145/81)	43.3 ± 11.8	82% women	20.9 ± 12.1	28% migraine with aura	2 m	≥50% reduction of migraine attacks	1 m	Acupuncture	Sham acupuncture	23	41.4%/17.3%
							≥50% reduction of migraine days	1 m, 4 m				
Xu 2020, China	150 (60/60/30)	36.3 ± 11.4	80% women	10.0 ± 5.0	–	2 m	Attacks per month	1 m, 2 m, 3 m	Acupuncture	Sham acupuncture/ Waitlist	2	8%/0/0
							Total migraine days per month					
Diener 2006, Germany	960 (313/339/308)	37.7 ± 10.5	83% women	16.7 ± 11.7	50% migraine with aura	6w	Total migraine days per month	3 m, 6 m	Acupuncture	Sham acupuncture / Waitlist	166	24.0%/23.6% /19.5%
Wang 2015, Australia	50 (26/24)	42.7 ± 14.1	74% women	19.7 ± 12.9	42% migraine with aura	5 m	Total migraine days per month	3 m, 12 m	Acupuncture	Sham acupuncture	3, 25*	NR
Zhao 2017, China	165 (83/82)	36.4 ± 14.2	76% women	9.2 ± 7.6	–	1 m	Total migraine days per month	1 m,2 m, 3 m, 4 m, 5 m	Acupuncture	Waitlist	0	6.0%/2.4%
							Attacks per month					
							VAS					
Vickers 2004, the United Kingdom	401 (205/196)	46.3 ± 10.4	85% women	21.6 ± 13.9	–	3 m	Total migraine days per month	9 m	Acupuncture	Waitlist	100	2.4%/0
Musil 2018, Czech Republic	86 (42/44)	46.1 ± 11.5	89% women	24.9 ± 13.6	–	3 m	Attacks per month	3 m, 9 m	Acupuncture	Waitlist	2	2.4%/0
							VAS	3 m				
Yang et al. (2018), China	42 (21/21)	33.0 ± 3.4	89% women	–	–	1 m	Total migraine days per month	3 m	Acupuncture	Flunarizine	3	33.3%/42.9%
							VAS					
Shu et al. (2017), China	120 (60/60)	47 ± 9	76% women	0.9 ± 0.5	–	1 m	VAS	3 m, 4 m, 5 m	Acupuncture	Flunarizine	3	1.7%/0
Cai et al. (2022), China	110 (55/55)	41.5 ± 9	60% women	11.1 ± 4.8	–	1 m	Total migraine days per month	1 m, 3 m, 6 m	Acupuncture	Flunarizine	0	1.7%/5.5%
							VAS					
Streng 2006, Germany	114 (59/55)	40.2 ± 11.0	84% women	–	90% migraine with aura	3 m	Total migraine days per month	3 m	Acupuncture	Metoprolol	26	15.3%/78.2%
							Attacks per month					

^*^Three patients dropped out at 3 months and 25 dropped out at 12 months.

### 3.2. Risk of bias assessment

**Figure 3:**
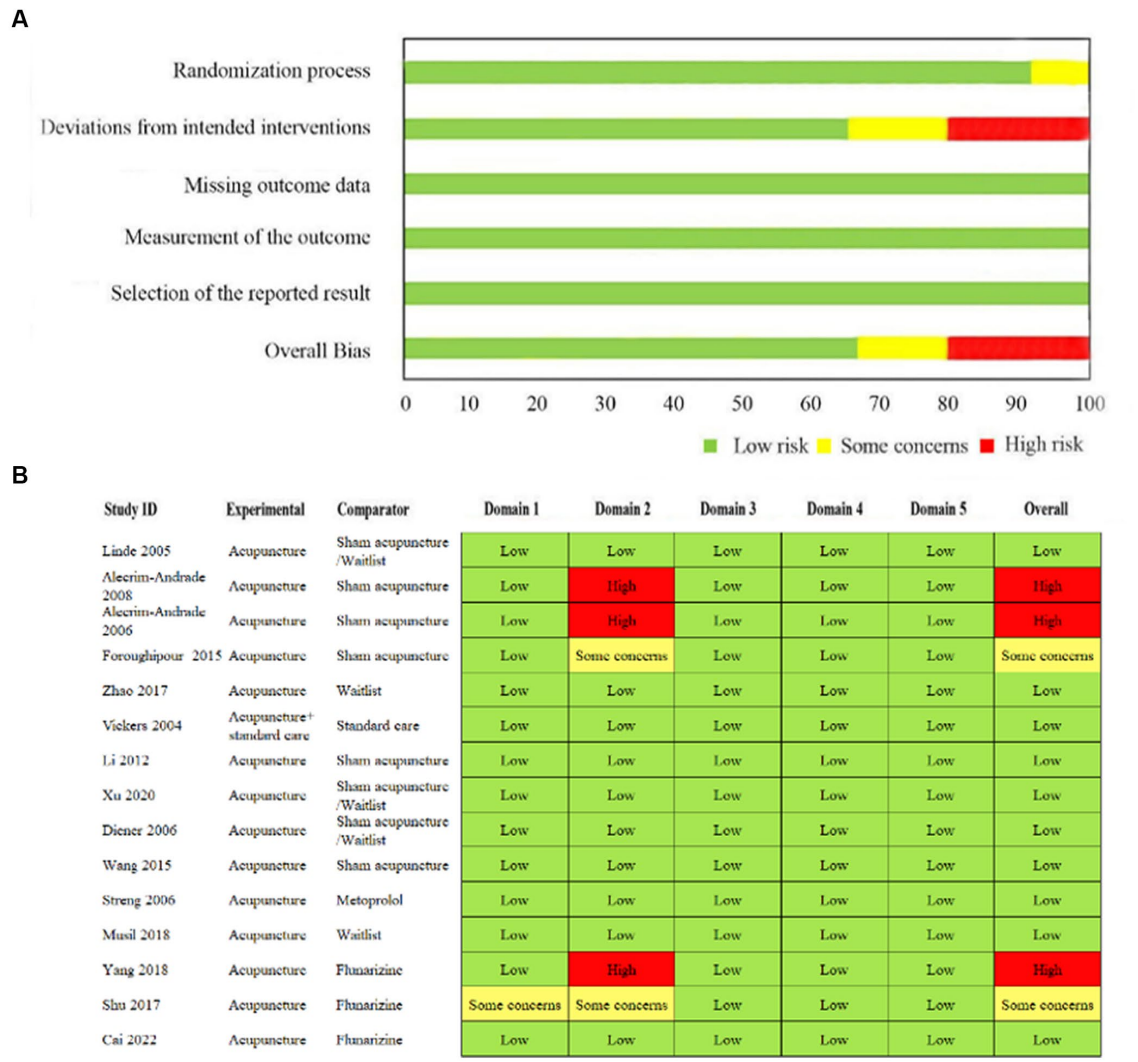
**(A)** Risk of bias graph presented as percentages. **(B)** Cochrane Risk of Bias 2.0 Tool of included studies.

Ten studies (Vickers et al., 2004; Linde et al., 2005; Diener et al., 2006; Streng et al., 2006; Li et al., 2012; Wang et al., 2015; Zhao et al., 2017; Musil et al., 2018; Xu et al., 2020; Cai et al., 2022) had a low RoB. One study (Shu et al., 2017) did not report the concealment of the allocation sequence. Five studies (Alecrim-Andrade et al., 2006, 2008; Foroughipour et al., 2014; Shu et al., 2017; Yang et al., 2018) did not sufficiently describe the process of intervention deviation. However, three studies (Alecrim-Andrade et al., 2006, 2008; Yang et al., 2018) with high dropout rates neither described the intervention deviation nor used intention-to-treat analysis to avoid the potential of a substantial impact on the result. Thus, one study (Shu et al., 2017) had a considerable RoB in the randomization process, three studies (Alecrim-Andrade et al., 2006, 2008; Yang et al., 2018) had high RoBs, and two studies (Foroughipour et al., 2014; Shu et al., 2017) had considerable RoBs in the domain of deviations from intended interventions (Figure 3).

### 3.3. Acupuncture versus sham acupuncture

**Figure 4:**
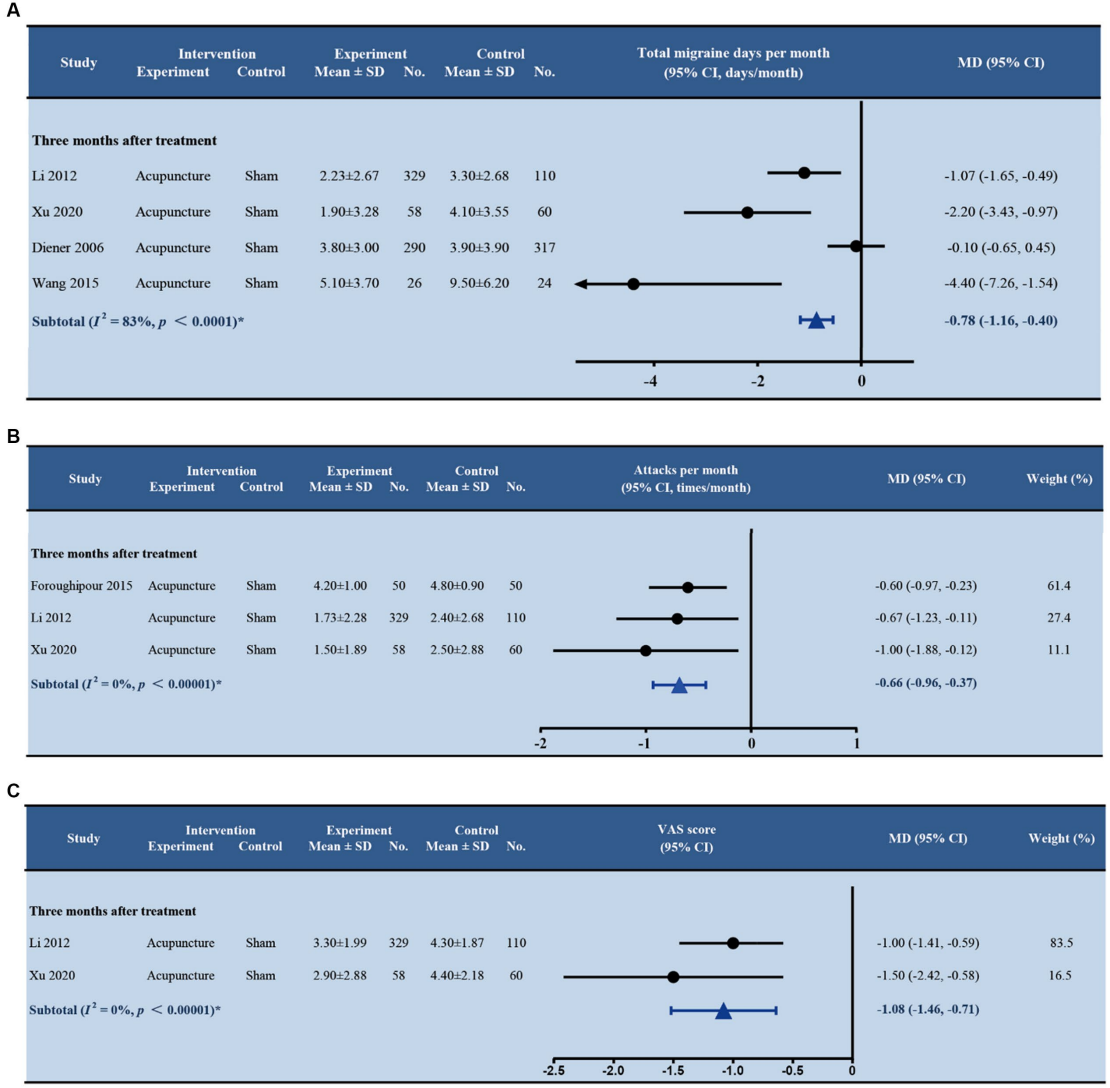
Forest plot of primary outcomes (acupuncture vs. sham acupuncture). **(A)** Total migraine days per month; **(B)** Attacks per month; **(C)** VAS score. SD, standard deviation; No., number of subjects; MD, mean difference; CI, confidence interval.

Eight studies (Linde et al., 2005; Alecrim-Andrade et al., 2006; Diener et al., 2006; Alecrim-Andrade et al., 2008; Li et al., 2012; Foroughipour et al., 2014; Wang et al., 2015; Xu et al., 2020) were found comparing acupuncture with sham acupuncture with follow-ups ranging from 1 to 12 months after treatment (Figure 4). Acupuncture reduced significantly more migraine attacks than sham acupuncture at 3 months after treatment (MD -0.66; 95% CI –0.96, −0.37; *p* < 0.001; and *I^2^* = 0%). Patients in the acupuncture group had significantly fewer days with migraine per month than in the sham acupuncture group at 3 months after treatment (MD -0.78; 95% CI –1.16, −0.40; *p* < 0.001). The random effect model was employed due to the heterogeneity among the four trials (*I^2^* = 83%). Two studies (Diener et al., 2006; Wang et al., 2015) reported that acupuncture was probably better than sham acupuncture in reducing the number of days with migraine per month at 6 and 12 months after treatment, separately, but the differences were not significant. No more evidence on the durable effect of acupuncture beyond 3 months was found (Supplementary Data Sheets S3, S4).

Three studies reported a reduction of pain intensity at 3 months after treatment. In Li et al. (2012) and Xu et al. (2020) studies, pain intensity was assessed by the VAS score. The minimal clinically important difference (MCID) in VAS score was defined as a 1.3 difference (Gallagher et al., 2001). The pooled estimate of VAS score at 3 months between acupuncture and sham acupuncture was significant (MD –1.08; 95% CI –1.46, −0.71; *p* < 0.001) in both trials but did not reach MCID (Figure 4). A study by Wang et al. (2015) employed a six-point Likert scale to assess pain intensity. Acupuncture reported a lower score than sham acupuncture at 3 months after treatment, but no significant difference was found (MD 0.3; 95% CI –0.5, 0.0; *p* = 0.087).

**Figure 5:**
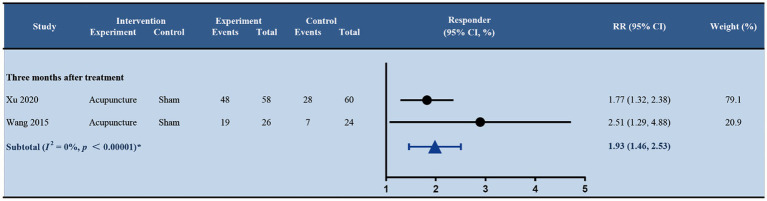
Forest plot of response rate (acupuncture vs. sham acupuncture). RR, relative risk.

Two studies (Wang et al., 2015; Xu et al., 2020) reported the response rate, defined as the proportion of patients with a reduction in the number of migraine days by 50% or more, at 3 months after treatment (Figure 5). Acupuncture reached a significantly higher response rate than sham acupuncture (RR 1.93; 95% CI 1.46, 2.53; *p* < 0.001; and *I*^2^ = 74%). Furthermore, in two trials by Alecrim-Andrade et al. (2006, 2008), patients in the acupuncture group experienced fewer migraine days with nausea as well as fewer migraine days with vomiting than in the sham acupuncture group at 6 months after treatment; however, no significant difference has been found.

### 3.4. Acupuncture versus waitlist

**Figure 6:**
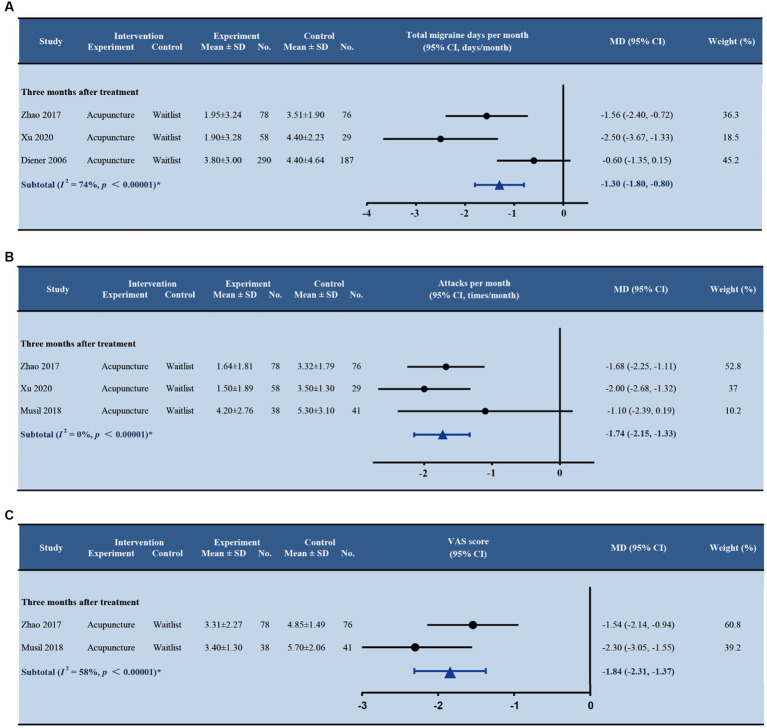
Forest plot of primary outcomes (acupuncture vs. waitlist). **(A)** Total migraine days per month; **(B)** Attacks per month; **(C)** VAS score. SD, standard deviation; No., number of subjects; MD, mean difference; CI, confidence interval.

Five studies (Vickers et al., 2004; Diener et al., 2006; Zhao et al., 2017; Musil et al., 2018; Xu et al., 2020) were found comparing acupuncture with waitlist with follow-ups ranging from 1 to 9 months after treatment. The durable effect of acupuncture sustained over 3 months after treatment is shown in Figure 6. The pooled estimated effect of three studies (Zhao et al., 2017; Musil et al., 2018; Xu et al., 2020) showed that acupuncture reduced significantly more migraine attacks than waitlist at 3 months (MD –1.74; 95% CI –2.15, −1.33; *p* < 0.001; *I*^2^ = 0%). For the number of days with migraine (Diener et al., 2006; Zhao et al., 2017; Xu et al., 2020), acupuncture also provided significantly more benefit than waitlist at 3 months after treatment (MD –1.30; 95% CI –1.80, −0.80; *p* < 0.001; and *I*^2^ = 74%). Four studies followed up with the patients for more than 3 months after treatment and found inconsistent results (Supplementary Data Sheets S5, S6). Two studies (Vickers et al., 2004; Zhao et al., 2017) reported significant between-group differences (at 4 and 5 months after treatment in Zhao’s study, and at 9 months after treatment in Vickers’ study), while the differences were not significant in another two studies (Diener et al., 2006; Musil et al., 2018) (at 6 months after treatment in Diener’s study, and at 9 months after treatment in Musil’s study).

Two trials (Zhao et al., 2017; Musil et al., 2018) comparing acupuncture with waitlist measured the VAS score at 3 months after treatment. We pooled the data from both studies and found a statistically significant difference between groups on VAS score (MD –1.84; 95% CI –2.31, −1.37; *p* < 0.001; and *I*^2^ = 58%), and the difference reached MCID. Only one study by Zhao et al. (2017) reported longer follow-ups. It seemed that the effect of acupuncture on alleviating pain still existed at 4 months after treatment (MD –1.66; 95% CI –2.24, −1.08; *p* < 0.001) and 5 months after treatment (MD –1.87; 95% CI –2.43, −1.31; *p* < 0.001), showing both statistically and clinically important differences (Supplementary Data Sheet S7).

Four studies reported the response rate for 3 months or more after treatment. Three out of the four trials (Vickers et al., 2004; Musil et al., 2018; Xu et al., 2020) suggested that acupuncture reached significantly higher rates of responders than waitlist at 3 months after treatment 64.7 (MD 64.7%; 95% CI 44.2, 85.1%; *p* < 0.001), 6 months after treatment (RR 2.26; 95% CI 1.44, 3.53; *p* = 0.0004), and 9 months after treatment (MD 15%; 95% CI 6, 25%; *p* = 0.02). However, one study (Diener et al., 2006) reported no significant difference between acupuncture and waitlist in response rate at 5 months after treatment (RR 1.14; 95% CI 0.92, 1.42; *p* = 0.22).

### 3.5. Acupuncture versus medication

#### 3.5.1. Acupuncture versus flunarizine

**Figure 7:**
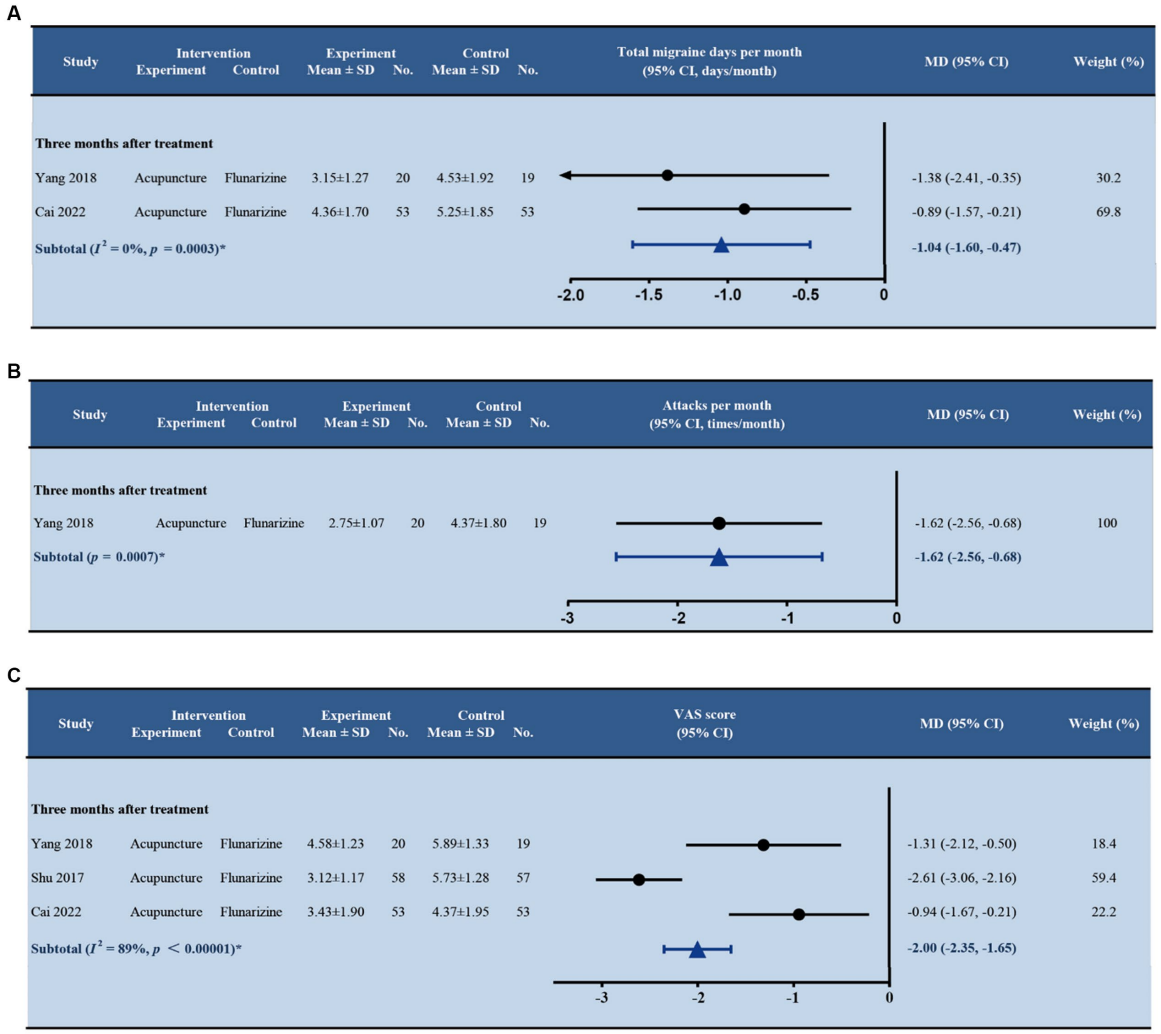
Forest plot of primary outcomes (acupuncture vs. flunarizine). **(A)** Total migraine days per month; **(B)** Attacks per month; **(C)** VAS score. SD, standard deviation; No., number of subjects; MD, mean difference; CI, confidence interval.

Three trials (Shu et al., 2017; Yang et al., 2018; Cai et al., 2022) comparing acupuncture with flunarizine for episodic migraine were analyzed (Supplementary Data Sheets S8–S10). Yang’s study (Yang et al., 2018) reported that acupuncture led to fewer migraine attacks per month than flunarizine with a statistically significant difference between groups (MD –1.62; 95% CI –2.56, −0.68; *p* < 0.001). For the number of days with migraine, we pooled the data from studies by Yang and Cai (Yang et al., 2018; Cai et al., 2022) and found a statistically significant difference between groups (MD –1.04; 95% CI –1.60, −0.47; *p* < 0.001), as shown in Figure 7. There was non-significant heterogeneity between the studies (*I^2^* = 0%), hence fixed effect model was adopted. Three studies (Shu et al., 2017; Yang et al., 2018; Cai et al., 2022) were found assessing pain intensity at 3 months after treatment. The pooled estimate of VAS score at 3 months between acupuncture and sham acupuncture was significant (MD –2.00; 95% CI –2.35, −1.65; *p* < 0.001) in both trials but did not reach MCID (Figure 7). The random effect model was employed due to the significant heterogeneity amongst the three trials (*I^2^* = 89%).

#### 3.5.2. Acupuncture versus metoprolol

A study by Streng et al. (2006) compared acupuncture with metoprolol in patients with episodic migraine (Supplementary Data Sheets S11, S12). The number of migraine attacks per month was significantly lower in the acupuncture group than in the metoprolol group at 3 months after acupuncture treatment (MD –0.90; 95% CI –1.42, −0.38; *p* < 0.001). However, the between-group difference in total migraine days per month was not significant (MD –1.00; 95% CI –2.19, 0.19; *p* = 0.1).

### 3.6. Adverse event

Overall, 13 studies reported information on adverse events. The rates of AEs in the acupuncture group were 1.7–41.4%, and those in the sham acupuncture group were reported by six of the 13 studies as 0–23.6% (Table 2). The main pattern of AEs was similar in both acupuncture and sham acupuncture groups, all reported as mild to moderate and not requiring special intervention. The most frequent type of AE was subcutaneous hemorrhage at needling sites. Also, some patients complained about pain in the puncture area (19 received acupuncture vs. 6 received sham acupuncture) and other AEs, including fatigue (6 vs. 1), palpitation (2 vs. 0), and ankle swelling (1 vs. 0). In addition, two studies (Vickers et al., 2004; Linde et al., 2005) reported a total of 23 cases of headache after treatment (17/383 in the acupuncture group vs. 6/112 in the sham acupuncture group).

## 4. Discussion

In this meta-analysis, 15 studies were analyzed to evaluate the durable effect of acupuncture for patients with episodic migraine. Acupuncture was significantly better than sham acupuncture, waitlist, and flunarizine in reducing the number of days with migraine per month and migraine attacks per month at 3 months after treatment. According to pooled estimates, acupuncture achieved a significant reduction in clinical importance in pain intensity measured by VAS score than waitlist and prophylactic drugs. Evidence on the pain-alleviating effect of acupuncture compared with sham acupuncture was inadequate.

As a worldwide prevalent life-span disorder, migraine negatively impacts patients’ quality of life and causes disability and comorbidity. We conducted this systematic review and meta-analysis to evaluate the durable effect of acupuncture for episodic migraine in response to the need to reduce migraine frequency, severity, and headache-related depression. Overall, the therapeutic successes of acupuncture for episodic migraine lasted for at least 3 months after discontinuation of treatment. Compared with waitlist or sham control, the response rate (≥50% reduction of migraine days) of acupuncture was reported to be significantly higher in most of the included studies. Previous literature also focused on the efficacy of acupuncture in comparison with pharmacological treatment in episodic migraine (Hesse et al., 1994; Allais et al., 2002; Diener et al., 2006; Streng et al., 2006; Wang et al., 2011; Facco et al., 2013; Zhang et al., 2020). The majority of these studies compared acupuncture with monotherapy as a prophylactic treatment. Recently, one prospective, randomized controlled trial compared acupuncture with the best prophylactic drugs for patients taking into consideration comorbidities (i.e., depression, insomnia, hypertension, etc.) and previous preventive treatment (Giannini et al., 2020). This trial showed that acupuncture was as effective as the most appropriate pharmacological treatment for migraine prophylaxis. On the total sample completing the treatment, 33.0 and 25.4% required prophylaxis therapy after 3 and 6 months, respectively, with a higher proportion in patients randomized to the pharmacological group (*n* = 19/46, 41.3% after T2; *n* = 8/46, 17.4% after T3) than those randomized to the acupuncture group (*n* = 15/57, 26.3% after T2; *n* = 7/57, 12.3% after T3). The improvements observed at the end of treatment persisted after therapy in 57.3% (59/103) after 3 months (T3) and in 38.8% (40/103) after 6 months (T4), especially in patients randomized to acupuncture treatment.

**Figure 8:**
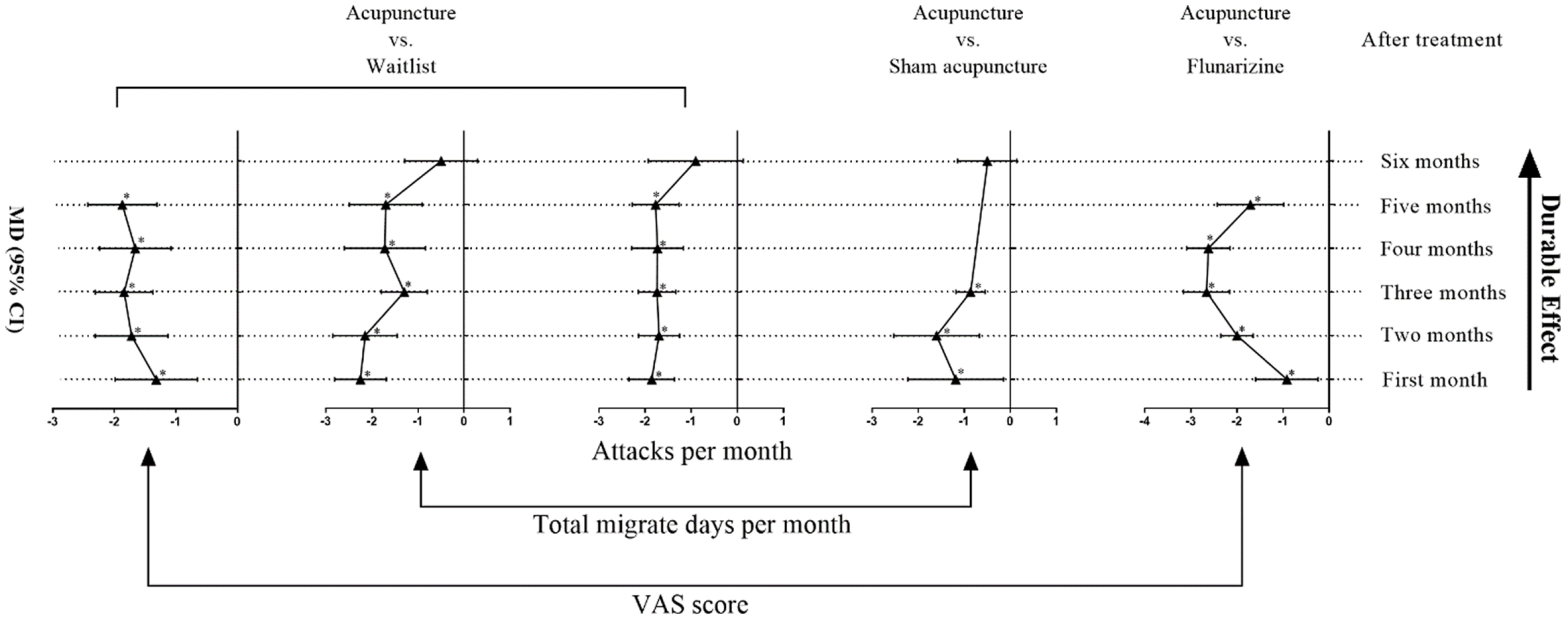
Schematic of the durable effect of acupuncture for episodic migraine.

However, current evidence was insufficient to reach a conclusive recommendation. According to results from current RCTs evaluating the durable effect of acupuncture for episodic migraine, no conclusion could be drawn about how the durable effect of acupuncture changes with time. Figure 8 shows a rough schematic of results from included studies. Besides, AEs of acupuncture rarely persist or appear newly after treatment discontinuation because the reported AEs were all mild to moderate and soon disappeared without particular intervention.

In the past decades, acupuncture has been pointed out as a valuable non-pharmacological tool in patients with migraine. In acupuncture research, true acupuncture is often compared with sham acupuncture. There are many different types of sham acupuncture intervention; these include lack of skin penetration by the needle, shallow penetration of the needle, insertion at points that are not traditional acupuncture points, or not achieving “deqi” which is an expected needling response (the subjective sensation of local warmth and paresthesia tenderness) that is considered an integral element of the healing process. Any intervention involving skin penetration cannot be considered an inert placebo. Sham acupuncture may still induce a wide range of peripheral, segmental, and central physiological responses to an unpredictable degree.

In this review, 11 out of 15 studies followed up patients for 3 months after treatment (73%), 4 studies followed up for 6 months after treatment (27%), and only one study followed up for 12 months after treatment (7%). Of the eight studies that evaluated the durable effects of acupuncture for episodic migraine compared with sham acupuncture, the results of the five studies with 3-month post-treatment follow-ups showed a significant reduction in the number of migraine attacks, the number of migraine days, and VAS score, in favor of acupuncture. However, the results of the only study that reported the primary outcome 6 months after treatment found no significant difference between acupuncture and sham acupuncture in reducing the number of migraine days (MD –0.50; 95% CI –1.14, 0.14; *p* = 0.13). Similarly, at 12 months after treatment, only one study with 50 patients (26/24) reported no significant difference in reducing the number of migraine days between acupuncture and sham acupuncture (MD –1.00; 95% CI –4.08 2.08; *p* = 0.52). Future studies with longer follow-up periods are required to evaluate the duration of acupuncture’s treatment effect after the completion of therapy.

According to the included studies above, patients received 8–15 treatments over 4–12 weeks and obtained durable therapeutic effects after treatment for at least 3 more months. Since the current therapies could barely provide sustained effects after drug withdrawal (Wöber et al., 1991), the durable effect of acupuncture showed potential advantages in decreasing the burden of migraine, improving the patient’s mood and quality of life (Dodick, 2018), and slowing the chronic evolutive process of migraine (May and Schulte, 2016; Andreou and Edvinsson, 2019). More high-quality clinical trials on the durable effect of acupuncture for migraine with longer follow-up periods are needed to investigate the benefits of acupuncture. Meanwhile, cost-effectiveness studies of acupuncture for migraine should consider the durable effect when measuring the time horizon.

Several previous reviews in the last decade evaluated the treatment effect of acupuncture for migraine, while only one systematic review collected follow-up outcomes after treatment. In the 2016 review by Linde et al. (2016), the authors pooled the follow-up outcomes at different time points together and reported significant post-treatment benefits on headache frequency and response rate (at least 50% frequency reduction) in favor of acupuncture compared with sham acupuncture or no acupuncture. Our results were consistent with the results of the previous review by Linde et al. (2016). Furthermore, our research clarified the durable effect of acupuncture 3 months after treatment and identified the pain-alleviating effect of acupuncture using the VAS score. Recently, a systematic review assessed the efficacy and safety of acupuncture for the prophylaxis of episodic or chronic migraine in adult patients compared to pharmacological treatment (Giovanardi et al., 2020), including nine randomized trials (1,484 patients). At the end of the intervention, the authors found a small reduction in favor of acupuncture for the number of days with migraine per month: (SMD: –0.37; 95% CI –1.64 to −0.11), and for response rate (RR: 1.46; 95% CI 1.16–1.84), a moderate effect in the reduction of pain intensity in favor of acupuncture (SMD: –0.36; 95% CI –0.60 to −0.13), and a large reduction in favor of acupuncture in both the dropout rate due to any reason (RR 0.39; 95% CI 0.18–0.84) and the dropout rate due to adverse event (RR 0.26; 95% CI 0.09–0.74). The quality of evidence was moderate for all these primary outcomes. These results seem partially in contrast with the results of the present review.

There are some limitations to this review. First, although most of the included studies had a low risk of bias, five studies still had a high or considerable risk of bias, causing potential heterogeneity. Second, the review was not able to assess the durable effect of acupuncture at follow-ups longer than three months after treatment due to a lack of data. Third, studies with different types of sham acupuncture (with or without penetration) and different drug dosages were all included, which may impact the pooled estimate effect. Therefore, the interpretation of the results of this review should be cautious.

## 5. Conclusion

Current studies suggested that acupuncture had a durable effect on episodic migraine for at least 3 months after treatment discontinuation. Acupuncture should be recommended to the migraine population, considering the rising global problem with medication-overuse headache (MOH) and the 15% non-responders to pharmacological management. Future clinical trials with robust methodological quality and longer follow-ups are needed to further investigate the durable effect of acupuncture.

## Data availability statement

The original contributions presented in the study are included in the article/Supplementary material, further inquiries can be directed to the corresponding author.

## Author contributions

HS and RM contributed equally to this manuscript. HS and ZL designed and conceptualized the study. HS, SG, and JF searched the databases, extracted data from included studies, and assessed risk of bias. HS, RM, and LZ contributed to the data analysis. HS and ZL contributed to the revising of the manuscript. All authors contributed to drafting the initial manuscript and approved the final version of this manuscript to be published.

## Conflict of interest

The authors declare that the research was conducted in the absence of any commercial or financial relationships that could be construed as a potential conflict of interest.

## Publisher’s note

All claims expressed in this article are solely those of the authors and do not necessarily represent those of their affiliated organizations, or those of the publisher, the editors and the reviewers. Any product that may be evaluated in this article, or claim that may be made by its manufacturer, is not guaranteed or endorsed by the publisher.
